# Aspergillomarasmine A inhibits metallo-β-lactamases by selectively sequestering Zn^2+^

**DOI:** 10.1016/j.jbc.2021.100918

**Published:** 2021-06-25

**Authors:** David Sychantha, Caitlyn M. Rotondo, Kamaleddin H.M.E. Tehrani, Nathaniel I. Martin, Gerard D. Wright

**Affiliations:** 1 David Braley Centre for Antibiotic Discovery, McMaster University, Hamilton, Ontario, Canada; 2 M.G. DeGroote Institute for Infectious Disease Research, McMaster University, Hamilton, Ontario, Canada; 3 Department of Biochemistry and Biomedical Sciences, McMaster University, Hamilton, Ontario, Canada; 4 Biological Chemistry Group, Institute of Biology Leiden, Leiden University, Leiden, The Netherlands

**Keywords:** antibiotic resistance, metallo-β-lactamase, zinc, inhibition mechanism, enzyme degradation, AMA, ayspergillomarasmine A, B7–Mal, biotin–maleimide, CAMHB, cation-adjusted Mueller Hinton broth, DM, N-[2-(dansylamino)ethyl]maleimide, ESI–MS, electrospray ionization–MS, FLAG, DYKDDDDK peptide, HRP, horseradish peroxidase, IMP, imipenemase, IP, immunoprecipitation, ITC, isothermal titration calorimetry, MBL, metallo-β-lactamase, NDM, New Delhi MBL, Ni–NTA, nickel–nitrilotriacetic acid, OM, outer membrane, PAR, 4-(2-pyridylazo)resorcinol, Phos, 5′ phosphorylated, PVDF, polyvinylidene difluoride, RNAP, *E. coli* RNA-polymerase subunit α, VIM, Verona integrin-encoded MBL

## Abstract

Class B metallo-β-lactamases (MBLs) are Zn^2+^-dependent enzymes that catalyze the hydrolysis of β-lactam antibiotics to confer resistance in bacteria. Several problematic groups of MBLs belong to subclass B1, including the binuclear New Delhi MBL (NDM), Verona integrin-encoded MBL, and imipenemase-type enzymes, which are responsible for widespread antibiotic resistance. Aspergillomarasmine A (AMA) is a natural aminopolycarboxylic acid that functions as an effective inhibitor of class B1 MBLs. The precise mechanism of action of AMA is not thoroughly understood, but it is known to inactivate MBLs by removing one catalytic Zn^2+^ cofactor. We investigated the kinetics of MBL inactivation in detail and report that AMA is a selective Zn^2+^ scavenger that indirectly inactivates NDM-1 by encouraging the dissociation of a metal cofactor. To further investigate the mechanism in living bacteria, we used an active site probe and showed that AMA causes the loss of a Zn^2+^ ion from a low-affinity binding site of NDM-1. Zn^2+^-depleted NDM-1 is rapidly degraded, contributing to the efficacy of AMA as a β-lactam potentiator. However, MBLs with higher metal affinity and stability such as NDM-6 and imipenemase-7 exhibit greater tolerance to AMA. These results indicate that the mechanism of AMA is broadly applicable to diverse Zn^2+^ chelators and highlight that leveraging Zn^2+^ availability can influence the survival of MBL-producing bacteria when they are exposed to β-lactam antibiotics.

Carbapenem-resistant organisms are a group of bacteria that pose a significant threat to human health (1). Their primary mechanism of antibiotic resistance involves the production of several different classes of β-lactamases, of which the Ambler class B metallo-β-lactamases (MBLs) are particularly problematic. The most concerning MBLs confer crossresistance to a broad range of β-lactam antibiotics (carbapenem, penicillin, and cephalosporin), including those coformulated with serine β-lactamase inhibitors (2, 3). MBLs are generally plasmid encoded, and their dissemination through horizontal gene transfer is frequent. Consequently, many MBL-producing members of Enterobacterales, *Pseudomonas* sp., and *Acinetobacter* sp. have emerged in the clinic (4). Infections caused by these resistant bacteria are difficult to treat and associated with high mortality among infected patients. There is an urgent need to control the spread of MBL-producing bacteria, develop new antibiotics to circumvent resistance, and salvage legacy β-lactam antibiotics with MBL inhibitors (5).

Three MBL subclasses (B1, B2, and B3) are produced by carbapenem-resistant organisms, but the most clinically important belong to subclass B1, including the New Delhi MBL (NDM), Verona integrin-encoded MBL (VIM), and imipenemase (IMP)-type enzymes (6). Class B1 MBLs are periplasmic Zn^2+^-dependent enzymes that hydrolyze all β-lactam antibiotic classes, except monobactams. The Zn^2+^ ions are essential to catalysis and occupy two sites (Zn_1_ and Zn_2_) within the substrate-binding cleft and bind cooperatively (7, 8, 9). The Zn^2+^ ions are crucial to producing the equivalent of a hydroxide ion that hydrolyzes the β-lactam ring of a bound substrate and stabilizing the reaction intermediates of the reaction (10, 11).

Aspergillomarasmine A (AMA) is an amino acid–derived fungal secondary metabolite that can reverse carbapenem resistance in animal models of infection (12). AMA itself is not antimicrobial but works in combination with β-lactam antibiotics by inhibiting MBL catalysis (Fig. 1). Based on existing *in vitro* studies, inhibition occurs through the ability of AMA to bind Zn^2+^ ions, which facilitates the efficient removal of a single metal ion from MBLs (12, 13). Recent work has shown that the inhibitory potency of AMA is not equal among different MBL families and alleles, as some are more sensitive to AMA than others (14). For example, reversal of resistance in NDM-1– and VIM-2–producing bacteria is more efficient than producers of IMP enzymes. This observation raises several important questions about the mechanism of AMA. Specifically, what main interactions, kinetic events, and enzymatic properties of class B1 enzymes (*i.e.*, Zn^2+^ affinity and protein stability) contribute to the efficacy of AMA, and can understanding these variables aid in the development of broad-spectrum MBL inhibitors?

**Figure 1:**
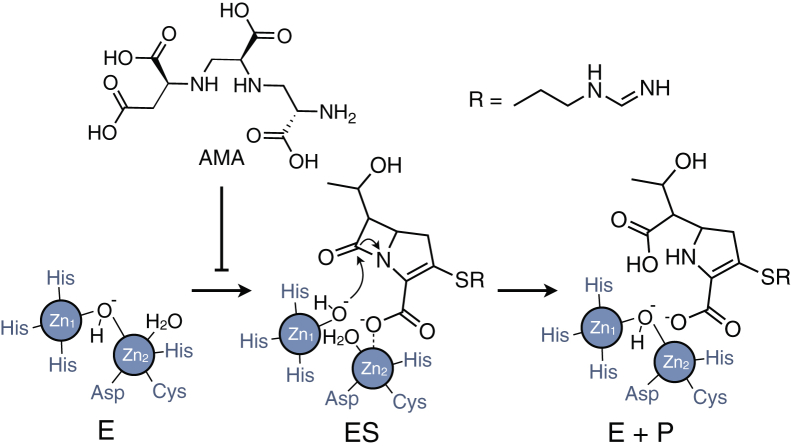
**Mechanism for the hydrolysis of imipenem by class B1 MBLs and inhibition by aspergillomarasmine A (AMA).** Catalysis by binuclear MBLs involves two Zn^2+^ cofactors bound in the Zn_1_ and Zn_2_ sites. E, ES, and P represent the free enzyme, enzyme–substrate complex, and product, respectively. AMA interferes MBL catalysis by removing one of the Zn^2+^ cofactors. MBL, metallo-β-lactamase.

Indeed, the usefulness of AMA as an MBL inhibitor has generated interest in its development for therapeutic use. Several methods for the total synthesis of AMA and analogs have been reported since 2016, including an efficient chemoenzymatic approach utilizing the reverse reaction of ethylenediamine-*N*,*N*′-disuccinic acid lyase (15, 16, 17, 18, 19, 20). New opportunities for the synthesis of AMA have also emerged from recent advances in understanding the biosynthesis of AMA, which revealed that only a single synthase is required for the production of AMA (21). AMA synthase catalyzes a one-step reaction from successive additions of *O*-phospho-l-Ser to l-Asp and can generate AMA derivatives when supplied with alternate substrates.

To inform the rational design and assessment of AMA analogs with improved inhibitory and/or pharmaceutical properties, we further characterized the activity of AMA and its mechanism of action both *in vitro* and in the context of the bacterial cell. Here, we show that the inhibitory activity of AMA is indirect, as it sequesters Zn^2+^ that has dissociated spontaneously from MBLs. In live bacteria, the potency of AMA is related to both Zn^2+^ affinity and protein stability of various MBLs, providing a mechanistic basis for its varied efficacy across MBL variants.

## Results

### Reversal of carbapenem resistance by AMA is determined by its binding selectivity toward Zn^2+^

**Figure 2:**
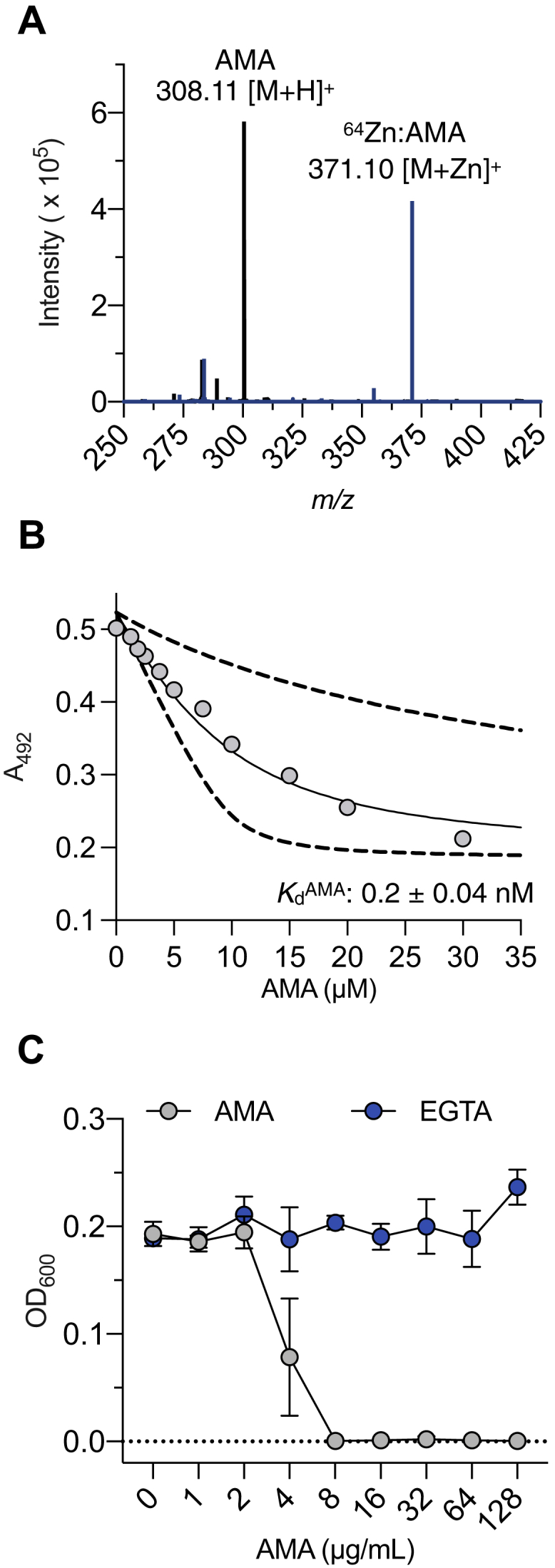
**NDM-1 inhibition by AMA is related to both its binding affinity and selectively for Zn**^**2+**^**.***A*, mass spectra of AMA in its free and complexed forms. *B*, representative ZnPAR_2_ (10 μM) absorbance in competition for Zn^2+^ with varying concentrations of AMA in 20 mM Hepes, 100 mM NaCl, and pH 7.4. The *solid line* represents the fit to a 1:1 Zn^2+^:AMA binding model, and *dashed lines* represent simulations with 10-fold higher and lower *K*_*d*_ values relative to the fitted mean. *C*, microdilution assay for the reversal of meropenem resistance in *Escherichia coli* producing NDM-1. AMA (*cyan*) or EGTA (*blue*) were titrated into cells in the presence of meropenem (2 μg⋅ml^−1^) and grown overnight at 37 °C. Absorbance values at 600 nm were recorded after 18 h of incubation. Data represent mean values of two replicates ± SD. AMA, aspergillomarasmine A; NDM-1, New Delhi MBL-1; PAR, 4-(2-pyridylazo)resorcinol.

The ability of AMA to bind to Zn^2+^ is central in its activity as an MBL inhibitor. However, the importance of the affinity and selectivity of AMA toward Zn^2+^ has not been analyzed in the context of complex biological fluids, which contain competitive metals that could influence its activity. We surveyed a panel of biologically relevant metal cations with isothermal titration calorimetry (ITC) analysis to address this knowledge gap. Our data showed that AMA did not interact appreciably with Mg^2+^ and Ca^2+^, whereas it did bind to Zn^2+^ and Ni^2+^ (Fig. S1). Previous work has also shown it to bind to Co^2+^ (13). Notably, the ITC binding assays revealed a very high-affinity interaction of AMA for Zn^2+^ as the isotherms resembled titrations performed with EDTA, which binds Zn^2+^ with extremely high affinity (*K*_*d*_^EDTA^ ≈10^−16^ M). However, ITC is not suitable for the precise determination of *K*_*d*_ values of high-affinity interactions (subnanomolar) because the change in heat capacity is not accurately measured (22). Consequently, we used a 4-(2-pyridylazo)resorcinol (PAR) competition assay to determine the strength of the AMA:Zn^2+^ interaction because it can determine dissociation constants over a wide range of affinities (10^−8^–10^−12^ M) (23). We first confirmed the expected 1:1 stoichiometry of the AMA–Zn^2+^ complex by electrospray ionization-MS (ESI-MS) analysis (Fig. 2*A*). Zn^2+^ affinity of AMA was then analyzed by titration with PAR as a chromogenic indicator, which showed that the *K*_*d*_^AMA^ = 0.2 ± 0.04 nM, under neutral conditions (20 mM Hepes–NaOH, 100 mM NaCl, and pH 7.4) (Fig. 2*B*). This value is similar to previously published values for the nonspecific metal chelator EGTA (*K*_*d*_^EGTA^ = 0.6 nM), for which we obtained a similar value (23). We note that the Zn^2+^ *K*_*d*_ value of AMA determined using this titration method differs by three orders of magnitude compared with our previously reported values determined by ITC (200 nM) (13). The discrepancy can be explained by the limitations of the ITC method noted previously.

Although AMA and EGTA have comparable affinity for Zn^2+^, their metal-binding profiles are distinct as the latter interacts with a broad spectrum of metals, whereas AMA is limited to Zn^2+^, Co^2+^, and Ni^2+^. We wondered whether such differences in selectivity affect their abilities to reverse carbapenem resistance in *Escherichia coli* producing NDM-1. As shown previously (14), AMA (8 μg⋅ml^−1^) reversed resistance and reduced the minimum inhibitory concentration of meropenem from 32 μg⋅ml^−1^ to its breakpoint of 2 μg⋅ml^−1^ in standard laboratory media (cation-adjusted Mueller Hinton broth [CAMHB]), which is rich in Mg^2+^ and Ca^2+^. In contrast, EGTA did not affect resistance under identical conditions, presumably because of competition with more abundant divalent cations (Fig. 2*C*). The effect of several metal ions on the inhibitory activities of AMA and EGTA was next investigated with *in vitro* enzyme assays using recombinant NDM-1, which was purified using conditions that yield enzyme containing 2 M equivalents of Zn^2+^ (12). Both AMA and EGTA inhibit >90% of NDM-1 activity at concentrations above 25 μM (Fig. S2). AMA or EGTA was then combined with equimolar amounts of Mg^2+^, Ca^2+^, Mn^2+^, Zn^2+^, Co^2+^, Fe^2+^, Fe^3+^, and Ni^2+^ and added to reaction mixtures. The inhibitory activity of AMA was not significantly affected by Mg^2+^, Ca^2+^, Mn^2+^, Fe^2+^, and Fe^3+^ and was blocked by Co^2+^, Ni^2+^, and Zn^2+^(Fig. S2*A*). On the other hand, EGTA activity diminished significantly in the presence of all the metals tested, except Mg^2+^ (Fig. S2*B*). These data demonstrate that the efficacy of AMA as a potentiator of meropenem is strongly related to both its high Zn^2+^ affinity and narrow metal selectivity, which overcomes competition with other biologically abundant metals.

### Spontaneous dissociation of Zn^2+^ from NDM-1 is the primary driver of inactivation

**Figure 3:**
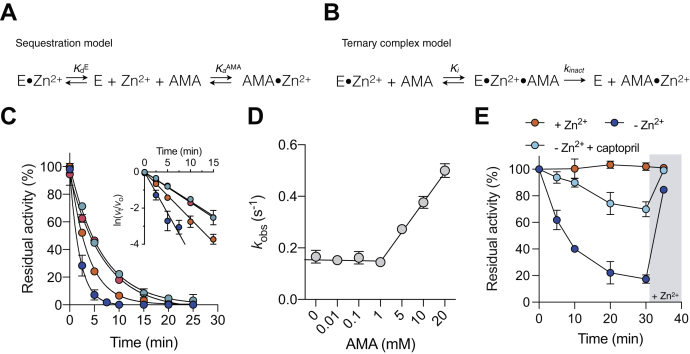
**Kinetic analyses reveal a dual mechanism of NDM-1 inhibition *in vitro*.***A*, minimal kinetic scheme for inhibition by sequestration, where E = MBL, *K*_*d*_^E^ = [E][Zn^2+^]/[E⋅Zn^2+^], and *K*_*a*_^AMA^ = [AMA⋅Zn^2+^]/[AMA][Zn^2+^]. *B*, minimal kinetic scheme for inhibition by ternary complex formation, where E = MBL, *K*_*i*_ = [E⋅Zn^2+^][AMA]/[EZn^2+^⋅AMA] and *k*_inact_ is the rate constant for the breakdown of the ternary complex to E and AMA⋅Zn^2+^. *C*, representative time courses for NDM-1 inhibition by AMA. Rates of inhibition were measured at fixed time intervals with 2 (*blue*), 5 (*orange*), 1 (*magenta*), and 0.1 mM (*cyan*) AMA. *Inset*, natural log transformation of the inhibition plot, where *v*_t_/*v*_o_ = residual activity. *D*, observed rate constants (*k*_obs_) determined from the previous panel relative to AMA concentration. *E*, spontaneous inactivation of NDM-1 in metal-free buffer (*blue*), metal-free buffer containing l-captopril (100 μM; *cyan*), and ZnSO_4_ (10 μM) supplemented buffer (*orange*). The gray bar denotes the addition of ZnSO_4_ to each mixture at *t* = 35 min. Metal-free buffer was produced by treating 25 mM Hepes–NaOH, 1% (v/v) with Chelex-100 resin. All data are representative of the mean value of two independent replicates, and error bars indicate SD. AMA, aspergillomarasmine A; MBL, metallo-β-lactamase; NDM-1, New Delhi MBL-1.

Chelators inhibit metalloenzymes through two main mechanisms (24). The chelator may either act indirectly and inhibit the enzyme by metal sequestration (Fig. 3*A*) and/or directly through the formation of a ternary complex, resulting in the removal of the active site metal ion(s) (Fig. 3*B*). Both models have been proposed for AMA; however, detailed enzymology of inhibition involving these mechanisms remains unclear (12, 13).

To begin characterizing the precise inhibitory mechanism of AMA, we determined the steady-state kinetics of NDM-1–catalyzed hydrolysis of nitrocefin over fixed time intervals and at different concentrations of AMA. Between 0.01 and 1 mM AMA, enzymatic inactivation was time dependent, occurred in one exponential phase, and followed first-order kinetics (Fig. 3*C*). The inhibition rate constant (*k*_obs_) was 0.16 ± 0.02 min^−1^, corresponding to a half-life (*t*_1/2_) of 4.1 min. Because the *k*_obs_ values were not dependent on AMA over a wide range of concentrations, this indicated that inhibition was not because of formation of an enzyme–AMA complex (Fig. 3*D*). Although such behavior was prominent at submillimolar AMA concentrations, we found that the rate of NDM-1 inhibition accelerated as AMA concentrations increased above 1 mM, resulting in a pseudo–first-order relationship that was nonsaturable. The slope represents the second-order rate constant (*k*_inact_/*K*_*i*_ = 18 ± 0.002 M^−1^⋅s^−1^). While this suggests a weakly formed ternary complex at high AMA concentrations, this activity is likely irrelevant to its mode of action in bacteria.

The addition of NDM-1 to assay buffer lacking AMA, which had been pretreated with Chelex-100 resin (*i.e.*, metal-free buffer), also resulted in the inactivation of NDM-1 (Fig. 3*E*). The rate of inactivation (*k*_obs_ = 0.16 min^−1^) is equivalent to the rate at low concentrations of AMA (<1 mM), suggesting that these inactivation events were mechanistically related. When AMA-inactivated NDM-1 (2 nM) was supplemented with ZnSO_4_ (10 μM), we found that its catalytic activity could be restored to at least 90% of its initial value (Fig. 3*E* and Fig. S3). This return of enzymatic activity occurred in ∼5 s (corresponding to the instrument's time lag), revealing that irreversible enzyme inactivation did not occur under the conditions tested with purified enzyme. Therefore, the observed inactivation constants at low enzyme and AMA concentrations approximate the rate of Zn^2+^ dissociation (*k*_off_) from NDM-1 and defines the rate-determining step for inactivation. To validate this, we incubated NDM-1 with the competitive inhibitor l-captopril (100 μM; *K*_*d*_ = 4 μM) (25) in metal-free buffer and monitored enzyme activity. l-Captopril was a poor inhibitor of NDM-1 when nitrocefin was used as substrate (IC_50_ = 418 μM), resulting in less than 10% inhibition and was disregarded in the analyses. The time-course assays showed that l-captopril reduced the rate of Zn^2+^ dissociation from NDM-1 (Fig. 3*E*). These observations are consistent with the known mechanism of inhibition where the thiol of l-captopril intercalates the two Zn^2+^ cofactors of NDM-1, competing with metal dissociation (26). Taken together, these data confirm that AMA operates through a metal scavenging mechanism of MBL inactivation.

### AMA captures Zn^2+^ that has spontaneously dissociated from the Zn_2_ site of NDM-1

Class B1 MBLs contain two Zn^2+^ cofactors bound in the Zn_1_ and Zn_2_ sites; the latter is reported to possess reduced Zn^2+^ binding affinity compared with Zn_1_ (27, 28, 29). Previous equilibrium dialysis studies showed that AMA treatment of NDM-1, VIM-2, and IMP-7 results in the loss of 1 M equivalent of Zn^2+^ from these enzymes (12, 13). Therefore, based on our inhibition data, we hypothesized that the Zn_2_ site of MBLs is involved in Zn^2+^ dissociation and subsequent AMA capture.

**Figure 4:**
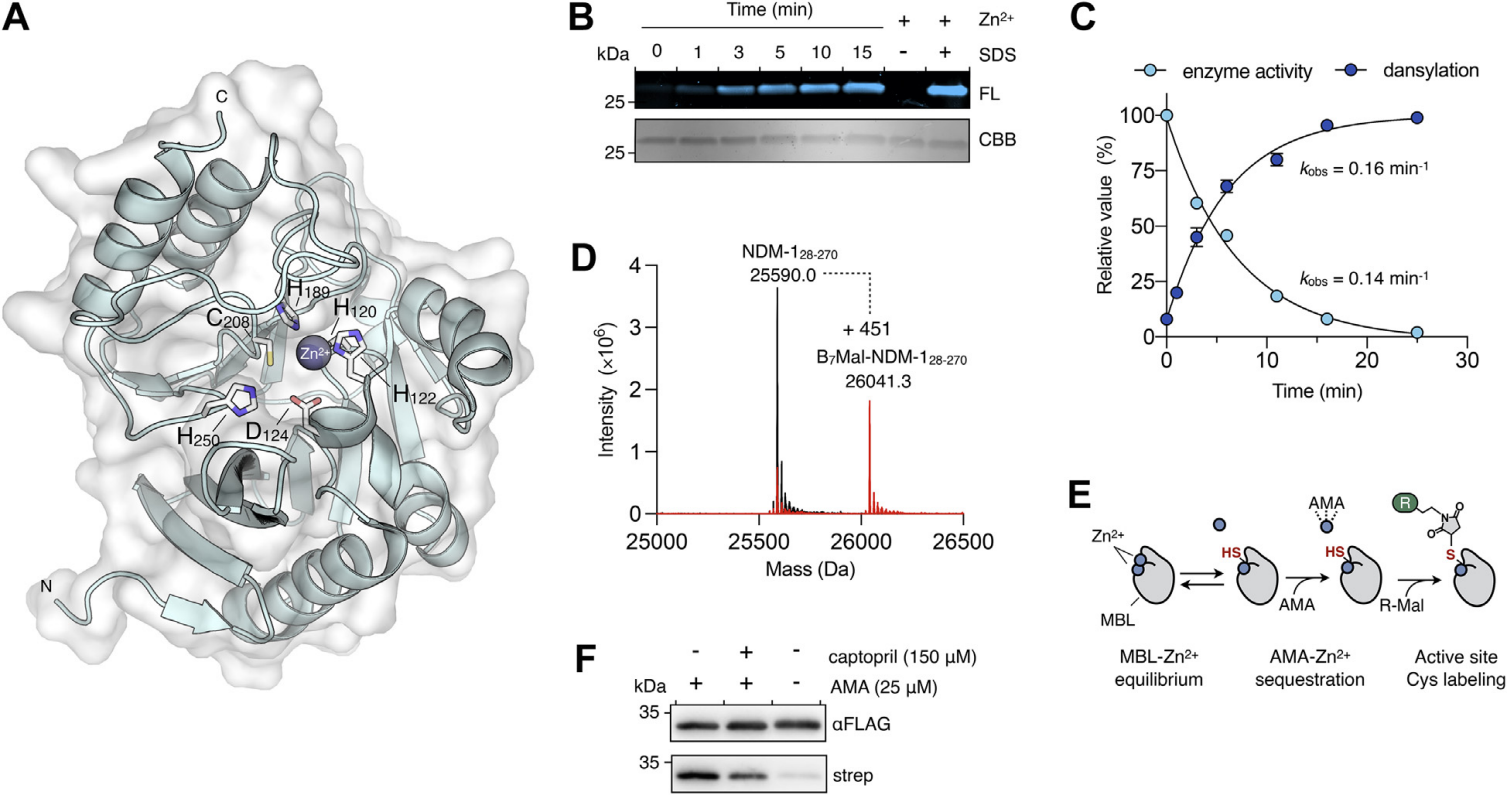
**Maleimide probes detect mononuclear MBLs through the action of AMA both *in vitro* and in live bacteria.***A*, structure of mono-Zn^2+^ NDM-1 illustrating the Zn^2+^ binding sites (Protein Data Bank ID: 3SFP). The Zn_1_ and Zn_2_ sites are defined by H_122_, H_120_, and H_189_ and D_124_, C_208_, and H_250_, respectively. *B*, representative *in vitro* time course of NDM-1 (1 μM) dansylation in assay buffer (25 mM Hepes–NaOH, 1% [v/v] PEG 4000, 10 μM ZnSO_4_, and pH 7.5) with AMA (0.1 mM) at 37 °C. At the end of the time course, ZnSO_4_ (0.11 mM) or ZnSO_4_ and SDS (2% w/v) were added to separate aliquots of the reaction. *Top*, SDS-PAGE analysis with fluorescence detection (FL) shows the increasing labeling of NDM-1 (1 μM) in the presence of DM (0.5 mM) and AMA (0.1 mM). NDM-1 is not labeled in the presence of ZnSO_4_ and entirely labeled when denatured with SDS. *Bottom*, Coomassie brilliant blue (CBB) staining of total protein. *C*, quantitative analysis of maleimide labeling (*blue*) in panel (*B*) relative to enzyme activity loss under the same conditions without DM present (*cyan*). The data are representative of two replicates, and the error bars represent SD. *D*, intact protein LC ESI–MS analyses of NDM-1 (1 μM) treated with B_7_–Mal following incubation (1 h) in buffer either lacking (*black trace*) or containing (*red trace*) AMA (0.5 mM). *E*, schematic diagram of a maleimide-based method to enable detection of mononuclear MBLs. AMA causes protein-bound Zn^2+^ to dissociate exposing the side chain of Cys_208_ that is involved in metal coordination in class B1 MBLs. A maleimide probe then modifies the Cys_208_ allowing for detection through reporter molecules (R = dansyl or biotin). *F*, *in situ* BM labeling and immunoprecipitation of NDM-1_FLAG_. *Escherichia coli*–producing NDM-1_FLAG_ was treated (15 min) with (+) or without (−) AMA (25 μM) and l-captopril (0.15 mM) for 15 min. Following this, cells were incubated with BM (0.5 mM; 15 min). Biotinylated protein and total NDM-1 were detected with streptavidin–HRP (strep) and anti-FLAG-HRP IgG (αFLAG), respectively. Panel *F* is representative of two independent experiments. AMA, aspergillomarasmine A; B_7_–Mal, biotin–maleimide; DM, *N*-[2-(dansylamino)ethyl]maleimide; ESI–MS, electrospray ionization–MS; FL, fluorescence; HRP, horseradish peroxidase; MBL, metallo-β-lactamase; NDM-1, New Delhi MBL-1.

The Zn_2_ site of class B1 MBLs contains a conserved cysteine residue (Cys_208_ in NDM-1) required for Zn^2+^ binding; consequently, we hypothesized that if the Zn_2_ site becomes vacant during inactivation, a free thiol would become exposed and be accessible to modification (Fig. 4*A*) (28, 30). We used the fluorescent thiol-specific probe, *N*-[2-(dansylamino)ethyl]maleimide (DM) in time-course assays in the presence of AMA to investigate this. The reaction was monitored by SDS-PAGE with fluorescence detection. NDM-1 showed time-dependent fluorescent labeling by DM in response to AMA (Fig. 4*B*). When ZnSO_4_ was added to NDM-1, the enzyme was protected from DM, whereas denaturation with SDS resulted in complete enzyme modification (Fig. 4*B*). The rate of Zn^2+^ dissociation from the Zn_2_ site could be quantified with the fluorescent labeling, which occurred in one phase, and it tracked with the rate of enzyme inactivation (Fig. 4*C*). Such inactivation/labeling behavior is consistent with a one-step dissociation mechanism involving the loss of a Zn^2+^ ion from the Zn_2_ site. Using this approach, the probe could not directly examine the metalation state of the Zn_1_ site; however, the kinetics suggest it could lose Zn^2+^ either concomitantly with the Zn_2_ site, or it remains in a Zn^2+^-bound state. As previous data showed that approximately 1 M equivalent of Zn^2+^ is lost to AMA, the latter likely represents the primary outcome (12, 13).

We also evaluated NDM-1 labeling *in vitro* with biotin–maleimide (B_7_–Mal), which is cell permeable and can be detected with electroblotting and chemiluminescence with streptavidin–horseradish peroxidase (HRP). After 30 min of AMA treatment, B_7_–Mal fully labeled NDM-1 within 1 min of incubation, which was in good agreement with our observations using DM (Fig. S4). The biotinylation of NDM-1 was 80% efficient as judged by intact protein ESI–MS (Fig. 4*D*) and occurred on Cys_208_ as determined by LC–MS/MS peptide mapping (Fig. S5). Although this implied that a subset (∼20%) of the NDM-1 protomers have Zn_2_ sites that are inaccessible to the maleimide probe, most of these sites appear to be vacant. Therefore, these results are generally consistent with a mechanism where dissociation of Zn^2+^ from Zn_2_ precedes sequestration by AMA (Fig. 4*E*).

### AMA sequesters Zn^2+^ from NDM-1 in live bacterial cells

The *in vitro* Zn^2+^ sequestration mechanism of AMA prompted us to reconcile its mechanism of action in live bacteria. To achieve this, we delivered B_7_–Mal into the bacterial periplasm. Here, labeling of mono-Zn^2+^ NDM-1 in its native environment could be detected by electroblotting whole outer membrane (OM) extracts of *E. coli*.

Because the OM of bacteria of the cell is exposed to atmospheric O_2_, it was anticipated that free thiols would be mostly oxidized, and background B_7_–Mal labeling and interference would be minimal. Unfortunately, AMA and B_7_–Mal treatment of *E. coli* resulted in extensive labeling of background thiols within the OM proteome and prevented clear identification of biotinylated NDM-1 (Fig. S6). We, therefore, engineered a DYKDDDDK peptide (FLAG) epitope at the C terminus of NDM-1 to facilitate immunoprecipitation (IP) and immunodetection with anti-FLAG antibodies. The epitope tag did not affect the resistance profile conferred by NDM-1 (Table S1). *E. coli* producing NDM-1_FLAG_ was treated with either AMA alone or together with l-captopril. An untreated sample served as the negative control. After 15 min of AMA treatment, B_7_–Mal was added to probe for mono-Zn^2+^-NDM-1_FLAG_, and the cells were left to equilibrate for an additional 15 min. Before cell lysis, residual B_7_–Mal was quenched with 2-mercaptoethanol so that labeling only occurred within live bacteria. IP analysis of NDM-1 from these samples showed that biotinylation was only faintly apparent in untreated cells. In contrast, AMA treatment resulted in significant labeling of NDM-1, which was partially blocked by l-captopril (Fig. 4*F*). These results were consistent with our *in vitro* data, showing that AMA sequesters Zn^2+^, which has dissociated from the Zn_2_ site of NDM-1 in the periplasm.

### *In situ* inhibition of MBLs by AMA is related to zinc affinity and stability of the enzyme

We recently demonstrated that the efficacy with which AMA potentiated β-lactam activity varied among different MBLs (14). Specifically, meropenem susceptibility was effectively restored by AMA in strains expressing *bla*_NDM-1_ and *bla*_VIM-2_ but less so for *bla*_NDM-6_ and *bla*_IMP-7_ (14). To better understand the mechanism behind this variability, we titrated AMA against live *E. coli* producing some of these MBLs, quantified changes in protein levels, and probed Zn^2+^ dissociation with B_7_–Mal.

**Figure 5:**
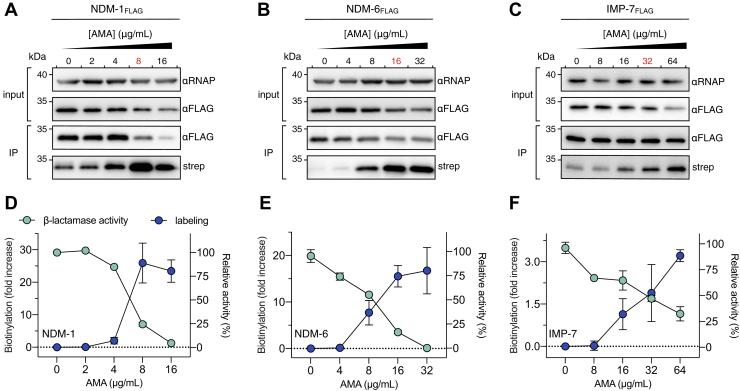
***In situ* inactivation of MBLs is related to Zn**^**2+**^**binding strength and protein stability.***A*–*C*, titration of AMA into cultures of live *Escherichia coli* producing *A*, NDM-1_FLAG_, *B*, NDM-6_FLAG_, or *C*, IMP-7_FLAG_ (15 min) followed by BM labeling (0.5 mM; 15 min). *E. coli* lysates (input; *top panel*) were analyzed by immunoblot analysis with anti-FLAG-HRP IgG (αFLAG) and anti-RNAP IgG (αRNAP) as a loading control. Immunoprecipitated MBLs (IP; *bottom panel*) were detected with αFLAG. The blot was reprobed with streptavidin–HRP (strep) to detect the extent of biotinylation of each protein (*bottom panel*). The AMA concentration colored *red* represents the amount needed to restore meropenem activity to its breakpoint concentration (2 μg⋅ml^−1^). Blots are representatives of at least two independent experiments. *D*–*F*, semiquantitative analysis of biotinylation normalized to the total amount of *D*, NDM-1, *E*, NDM-6, and *F*, IMP-7 (*cyan*; *left axes*). Residual β-lactamase activity of the cell lysates prior to IP analysis (input) was measured with the substrate nitrocefin (*blue*; *right axes*). In all experiments, data are represented by at least two independent biological replicates; data represent mean values ± SD. AMA, aspergillomarasmine A; HRP, horseradish peroxidase; IMP, imipenemase; IP, immunoprecipitation; MBL, metallo-β-lactamase; NDM, New Delhi MBL.

To facilitate this study, NDM-6 and IMP-7 were also engineered with C-terminal FLAG tags, which did not affect the resistance profiles of these MBLs in *E. coli* (Table S1). Bacteria were treated with varying amounts of AMA and a fixed amount of B_7_–Mal. The total protein levels of each MBL were subsequently determined by immunoblotting cell lysates with anti-FLAG antibodies. We found that NDM-1 levels decreased in response to AMA between 8 and 16 μg⋅ml^−1^ (Fig. 5*A*). In comparison, NDM-6 was more stable as its levels dropped more gradually at AMA concentrations between 16 and 32 μg⋅ml^−1^ (Fig. 5*B*). IMP-7 levels were the most stable and changed very little over the concentrations of AMA tested (Fig. 5*C*). To rule out the possibility that the probe introduced these stability defects, MBL levels were also determined in cells that were not treated with the B_7_–Mal. The levels were determined immediately following AMA treatment by lysing the cells in SDS-PAGE loading buffer, followed by immunoblotting. While the levels of NDM-1 and IMP-7 were similar to those containing probe under such conditions, NDM-6 appeared slightly more stable. The discrepancy between the NDM-6 data could be explained by the time dependence of degradation, augmented by the lengthy lysis and IP procedure following labeling (Fig. S7). Overall, these data are consistent with previous results showing that Zn^2+^ removal accelerates protein degradation in bacteria, and it is MBL specific (31).

IP analyses to quantify B_7_–Mal labeling were normalized to the total amount of each protein, as judged by immunoblotting. The data showed that the labeling profile of NDM-1 had a positive sigmoidal relationship to AMA concentration. This profile tracked with the decrease in residual β-lactamase activity in cell lysates of the treated bacteria as detected with substrate nitrocefin (Fig. 5*D*). Both the reduction of β-lactamase activity and the increase in B_7_–Mal labeling began to plateau at an AMA concentration of 8 μg⋅ml^−1^ until activity became undetectable at 16 μg⋅ml^−1^. (Fig. 5*D*). A similar profile was observed with NDM-6, except protein labeling and β-lactamase activity only began to plateau at 16 μg⋅ml^−1^ of AMA (Fig. 5*E*). In contrast, this relationship occurred more gradually with IMP-7. The inhibition profile was linear relative to AMA concentration, and enzyme activity was never wholly abolished as 35% residual activity was observed at 64 μg⋅ml^−1^ (Fig. 5*F*). These data suggest that enzymes with higher affinity Zn_2_ sites resist the action of AMA.

**Figure 6:**
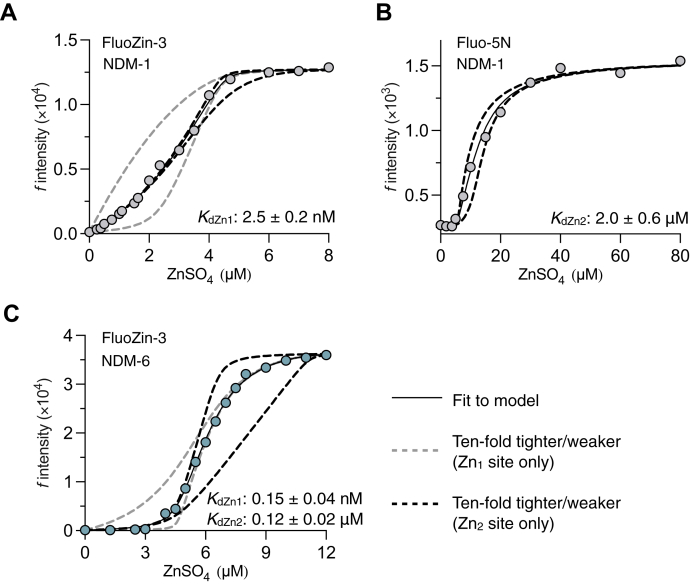
**Zn**^**2+**^**affinities of NDM-1 and NDM-6.***A*, representative fluorescence of FluoZin-3 (2 μM) in competition for Zn^2+^ with NDM-1 (2.4 μM). *B*, representative fluorescence of Fluo-5N (1 μM) in competition for Zn^2+^ with NDM-1 (5.5 μM). *C*, representative fluorescence of FluoZin-3 (2 μM) in competition for Zn^2+^ with NDM-6 (4.6 μM). The *solid line* represents the fit to a 2:1 Zn^2+^:MBL binding model, and *dashed lines* represent simulations with 10-fold higher and lower *K*_*d*_ values relative to the fitted mean when either the Zn_1_ (*gray*) or Zn_2_ (*black*) *K*_*d*_ values are fixed. All assays were performed using 20 mM Hepes–NaOH, 100 mM NaCl, and pH 7.5 in duplicate. MBL, metallo-β-lactamase; NDM, New Delhi MBL.

In light of the aforementioned observations, we determined the Zn^2+^ binding constants of NDM-1 and NDM-6. IMP-7 was excluded from this experiment because apoenzyme could not be reliably produced, as described previously (13). The affinities of the Zn_1_ and Zn_2_ sites from NDM-1 were determined by competition with the fluorescent probes FluoZin-3 (*K*_*d*_ = 9.1 nM) and Fluo-5N (*K*_*d*_ = 3.1 μM; Fig. S8), respectively (Fig. 6, *A* and *B*). Fluo-5N was chosen because competition with FluoZin-3 could not accurately estimate the Zn^2+^ binding constant of the low-affinity Zn_2_ site. The affinities of both sites from NDM-6 were both determined by competition with FluoZin-3 (Fig. 6*C*). These data showed that Zn^2+^ has a 20-fold weaker affinity for NDM-1 than NDM-6 and was reflected in both binding sites. Although the Zn^2+^ affinity for IMP-7 was not determined here, we suspect that the affinity is relatively high, as was shown previously for IMP-1 (32). Therefore, both Zn^2+^ affinity and protein stability characterize the variations in susceptibility of different MBLs to AMA.

## Discussion

Here, we present a comprehensive study demonstrating that the molecular mechanism of AMA affects MBLs indirectly by sequestering free Zn^2+^ ions. Withholding Zn^2+^ perturbs the equilibrium for subsequent formation of binuclear MBL enzymes causing practically irreversible Zn^2+^ dissociation concomitant with activity loss *in vivo*. Consequently, low-stability MBLs, such as NDM-1, are degraded in the periplasm of *E. coli*. In general, this inhibition mechanism could be broadly applied to many chelators. There are, however, differences regarding the metal selectivity and affinity of AMA, which provide it with some advantages.

AMA is markedly different from broad-spectrum chelators because it is well tolerated in animals (intravenous lethal dose, 50% = 160 mg⋅kg^−1^ in mice) (12). The narrow metal selectivity of AMA may significantly curtail toxicity, as Zn^2 +^ ions are preferred over other biologically abundant metal cations (*i.e.*, Mg^2+^, Ca^2+^, and Mn^2+^). Precedent for the low toxicity of AMA is supported by the long-term clinical use of selective chelators for treating iron overload. For example, the approved Fe^3+^ chelator deferoxamine is a natural product from *Streptomyces pilosus* with an appreciable affinity for Zn^2+^ (*K*_*d*_ = 10^−11^ M) (33). In this regard, it stands to reason that short-term treatment of bacterial infections using AMA may be tolerated well in humans. Animal models have shown that AMA can reverse resistance with only a single dose when combined with meropenem.

The Zn^2+^ dissociation constants of most clinically relevant MBLs are in the low micromolar to nanomolar range (14). This property of such problematic MBLs makes them particularly susceptible to metal sequestration. Hence, the subnanomolar Zn^2+^ affinity of AMA allows it to outcompete MBLs and agree with previous equilibrium dialysis and NMR studies probing the interactions of AMAs with NDM-1, VIM-2, and IMP-7 (13). In contrast, many human Zn^2+^-dependent enzymes, such as carbonic anhydrase, have very high affinities for Zn^2+^ with equilibrium constants ranging from picomolar to femtomolar affinity, resulting in dissociation half-lives, which range from days to months (24). Therefore, the binding strength of AMA is conveniently suited for MBL inactivation while minimizing off-target effects that would occur as a result of inhibiting high-affinity host metalloproteins.

There are drawbacks to the mechanism of AMA because of environmental selection for MBLs with improved enzyme activity under Zn^2+^ limiting conditions (25, 34). The ongoing evolution of Zn^2+^ affinity is well characterized in NDM-type MBLs, of which more than 20 novel allelic variants have arisen within the last decade. Each new NDM allele has gained at least one point mutation that increases Zn^2+^ affinity and protein stability. As shown here, both the Zn^2+^ binding sites of NDM-6 have 20-fold higher affinity relative to those in NDM-1 (14, 25). This contrasts with previous work, which proposed that the A233V substitution of NDM-6 was only responsible for improved stability. Indeed, both the enhanced metal affinity and protein stability corresponds with the need to use more AMA to restore meropenem sensitivity in *E. coli*. This is consistent with our *in situ* Cys_208_ labeling experiments, which show that NDM-6 can compete for metal ions more effectively.

This work provides complementary data derived from *in vitro* and *in situ* enzymatic analyses to support a mechanism where AMA inhibits MBLs through Zn^2+^ sequestration. We achieved this using both new and conventional techniques to analyze important features of MBL function that are often neglected in inhibitor characterization. The shortcomings of MBL inhibition through metal chelation by AMA were also illuminated by this study, highlighting a desire to develop MBL active site–targeted AMA analogs.

## Experimental procedures

### Bacterial strains and growth conditions

To evaluate β-lactam resistance *in vitro*, *E. coli* BW25113 was used as a model organism for *bla*_NDM-1_, *bla*_NDM-6_, *bla*_VIM-2_, or *bla*_IMP-7_ expression. *E. coli* TOP10 was used for the construction, maintenance, and propagation of plasmid DNA, and *E. coli* BL21(DE3) was used for protein production. Bacteria were routinely grown in liquid culture under aerobic conditions at 37 °C using either LB (Lennox formulation) or CAHMB supplemented with 50 μg⋅ml^−1^ kanamycin when appropriate. LB agar containing 50 μg⋅ml^−1^ kanamycin or an appropriate β-lactam antibiotic was used for growth on solid media.

### DNA manipulation

Plasmids containing *bla*_NDM-1_, *bla*_NDM-6_, *bla*_VIM-2_, and *bla*_IMP-7_ for the overproduction of MBLs were described in our previous work (14). For constitutive low-copy MBL expression, pGDP2 containing *bla*_NDM-1_, *bla*_NDM-6_, *bla*_VIM-2_, and *bla*_IMP-7_ were also obtained from our previous studies. C-terminal DYKDDDDK (FLAG)-tagged variants of NDM-1, NDM-6, and IMP-7 were engineered through whole-plasmid PCR amplification of existing pGDP2-based constructs with 5′ phosphorylated (Phos) primers. The resultant linear DNA was circularized by blunt-end ligation using T4 DNA ligase. PCR amplification was carried out with the universal forward primer 01-FLAG (5′ Phos-GATGACGACAAGTGAAAGCTTGCGGCCGCA) and gene-specific reverse primers 02-NDM-1_FLAG_ (5′ Phos-GTCTTTGTAGTCGCGCAGCTTGTCGGCCAT) for *bla*_NDM-1_ and *bla*_NDM-6_, 02-VIM-2_FLAG_ (5′ Phos-GTCTTTGTAGTCCTCAACGACTGAGCGATT) for *bla*_VIM-2_, and 02-IMP-7_FLAG_ (5′ Phos-GTCTTTGTAGTCGTTACTTGGTTTTGATAG) for *bla*_IMP-7_.

### Antibiotic susceptibility and potentiation assays

Meropenem susceptibility assays for determination of minimum inhibitory concentration values were performed in CAMHB using the microdilution method in 96-well round-bottom plates (Sarstedt) as recommended by the Clinical and Laboratory Standards Institute. Meropenem potentiation assays were carried out in a similar format, except the meropenem concentration was held constant at its European Committee on Antimicrobial Susceptibility Testing breakpoint value (2 μg⋅ml^−1^), and AMA or EGTA concentrations were varied (0.5, 1, 2, 4, 8, 12, 16, 24, 32, and 64 μg⋅ml^−1^). The concentration of potentiator required to restore the sensitivity of MBL-producing bacteria to meropenem represents the rescue concentration. Bioassays were incubated statistically for 20 h at 37 °C and were read spectrophotometrically at a wavelength of 600 nm using a BioTek Synergy H1 microplate reader (BioTek). All assays were performed with at least two replicates.

### Protein production and purification

As described previously, NDM-1 and NDM-6 were engineered in frame with an N-terminal His_6_-SUMO tag in pE-SUMO, whereas VIM-2 and IMP-7 were engineered with N-terminal His_6_ tags in pET-28b. All enzymes were engineered without their native N-terminal signal sequences. The production of MBLs was carried out using the expression strain *E. coli* BL21 in LB. Expression was induced at an absorbance of 0.6 at 600 nm with IPTG (1 mM), following which the cells were incubated at 16 °C overnight. Subsequently, the cells were harvested (5000*g*, 15 min), resuspended (50 mM Hepes, 300 mM NaCl, 5 mM imidazole, 100 μM ZnSO_4_, and pH 7.5), lysed by sonication (interval of 8 s for 8 min) using a Microson XL-2000 ultrasonic liquid processor (Qsonica), centrifuged to remove debris (15,000*g*, 15 min), and the cleared supernatant incubated with nickel–nitrilotriacetic acid (Ni–NTA) resin (Pierce; 1 h, 4 °C). The Ni–NTA resin was subsequently washed, and proteins were eluted with imidazole (250 mM). Pure fractions were pooled and buffer exchanged by dialysis into storage buffer (25 mM Hepes, 100 mM NaCl, 100 μM ZnSO4, and pH 7.5). SUMO-tag and His_6_-tag removal was achieved with Ulp-1 or thrombin proteases, respectively. Each digest was subsequently incubated with Ni–NTA resin (30 min; 4 °C), and untagged protein was recovered in the supernatant. Each protein was obtained at >95% purity as judged by SDS-PAGE.

### Enzyme kinetics

Routine determination of enzymatic activity was conducted by measuring β-lactam hydrolysis spectrophotometrically at 485 nm using a saturating amount of nitrocefin (30 μM) in assay buffer (25 mM Hepes–NaOH, 10 μM ZnSO_4_, and pH 7.5). Reactions were performed in a clear flat-bottom 96-well plate at 25 °C with a final assay volume of 200 μl, initiated by addition of substrate, and monitored with a BioTek Synergy H1 microplate reader over 2 min. NDM-1, NDM-6, and IMP-7 were used at working concentrations of 2, 2, and 10 nM, respectively. All reactions were performed in duplicate unless otherwise stated.

To determine the spontaneous rate of NDM-1 inactivation in metal-free buffer, a dilution assay was employed where enzyme (5 μM) in storage buffer was added to inactivation buffer (Chelex-100–treated 25 mM Hepes–NaOH, pH 7.5, 1% [v/v] PEG 4000; 37 °C), diluting it to its working concentration (10^3^-fold dilution). The enzyme activity was immediately measured by removing an aliquot and adding it to nitrocefin, followed by periodic measurements over 25 min. Enzyme-catalyzed nitrocefin hydrolysis reactions were measured for 2 min, over which rates of catalysis remained steady state and linear. In a parallel experiment, ZnSO_4_ (10 μM) was included in the inactivation buffer to serve as an enzyme stability control. The effect of a competitive inhibitor on the spontaneous inactivation of NDM-1 was achieved by including 100 μM l-captopril in the inactivation buffer, which allows for NDM-1 to retain ∼90% of its activity toward nitrocefin. The concentration was chosen based on dose-response experiments involving l-captopril (half maximal inhibitory concentration [IC_50_] = 420 μM) with nitrocefin as a substrate under the experimental conditions used in this study.

To determine the time dependence of AMA inhibition, this inhibitor was included in the inactivation buffer at varying concentrations (20, 10, 5, 1, 0.1, and 0.01 mM). Pseudo–first-order rate constants for inhibition (*k*_obs_) and half-lives (*t*_1/2_) at each AMA concentration were determined using nonlinear regression analysis with the one-phase decay function in GraphPad Prism 8 (GraphPad).

### Inactivation and labeling of NDM-1 *in vitro*

To label enzyme with *N*-[2-(dansylamino)ethyl]maleimide (Sigma; catalog no. 39326), AMA was added to NDM-1 (10 μM; prewarmed to 37 °C) in storage buffer at a final concentration of 0.5 mM. An aliquot (10 μl) was immediately removed and diluted in inactivation buffer (40 μl) containing DM (0.5 mM) for labeling. Following incubation with DM (1 min, 37 °C), the reaction was quenched with SDS-PAGE loading dye containing 0.5% (v/v) 2-mercaptoethanol (50 μl). Subsequently, additional aliquots were periodically removed and treated in the same manner over 20 min. The final aliquot was diluted in inactivation buffer containing both ZnSO_4_ (110 μM) before the addition of DM. An aliquot was diluted in storage buffer containing both DM and SDS (2%) before quenching to serve as a positive control. Each quenched aliquot was subsequently heated at 80 °C for 5 min and analyzed by SDS-PAGE. Fluorescence detection of DM-labeled enzyme was achieved with UV transillumination and a 590/110 nm filter on a ChemiDoc MP Imaging System (Bio-Rad). The gel was subsequently stained with Coomassie brilliant blue to determine the total amount of protein. Fluorescence intensity was quantified using ImageLab software (Bio-Rad) and normalized to the positive control. The *k*_obs_ of labeling was analyzed using the one-phase association function in GraphPad Prism 8.

Enzyme labeling with biotin–maleimide (Sigma; catalog no. B1267) was carried out similarly as described previously, except that only a single time point was taken after 20 min. Total protein was analyzed by SDS-PAGE, and labeling was assessed by Western blot on polyvinylidene difluoride (PVDF) membrane. Detection of B_7_–Mal labeled enzyme was achieved by probing the membrane with streptavidin–HRP and chemiluminescence using a ChemiDoc MP Imaging System.

### Immunodetection of FLAG-tagged MBLs

To determine the effect of AMA on the total protein levels of FLAG-tagged MBLs, overnight cultures were diluted in fresh CAMHB broth (1/100) and incubated at 37 °C until an absorbance of 0.8 at 600 nm was reached. Cells were then treated with varying amounts of AMA (32, 16, 8, 4, and 2 μg⋅ml^−1^) and incubated for an additional 30 min. Subsequently, cells were collected by centrifugation, resuspended in SDS-PAGE loading dye, and heated at 95 °C for 15 min. The samples were centrifuged (15,000*g*, 10min, room temperature) and analyzed by SDS-PAGE and Western blotting on PVDF membranes. The membrane was blocked with 5% nonfat skim milk, probed for FLAG-tagged MBLs with mouse-derived anti-DYKDDDDK (FLAG) IgG2b conjugated to HRP (GenScript; 1:5000 dilution), and were detected with chemiluminescence with a ChemiDoc MP Imaging System. The membrane was stripped and reprobed with mouse-derived anti-*E. coli* RNA-polymerase subunit α (RNAP) IgG1 (BioLegend; 1:5000 dilution) as a loading control. Mouse-derived anti-RNAP IgG1 was detected with rabbit antimouse-IgG, HRP-linked secondary antibodies (abcam; 1:5000 dilution), and chemiluminescence as described previously. Semiquantitative analysis of FLAG-tagged MBLs was carried out by integrating band intensity using ImageLab software and normalized to the signal obtained from RNAP.

### *In situ* labeling and IP

Overnight cultures of *E. coli* producing either NDM-1_FLAG_, NDM-6_FLAG_, or IMP-7_FLAG_ in CAMHB were diluted (1/100) in fresh CAMHB (5 ml) and grown until an absorbance of 0.8 at 600 nm was reached. At this point, AMA (25 μM), and l-captopril (150 μM) were added to the cultures and incubated for 15 min at 37 °C. A negative control was prepared in parallel without the addition of inhibitors. Subsequently, B_7_–Mal (0.5 mM) was added, and the cells were incubated for an additional 15 min at 37 °C. Unreacted B_7_–Mal was quenched with 1% (v/v) 2-mercaptoethanol for 5 min, and the cells were collected and washed three times by centrifugation (5000*g*, 15 min, 4 °C) with cold binding buffer (10 ml; 50 mM Tris, 150 mM NaCl, and pH 8.0). The resulting cell pellet was frozen in liquid nitrogen, resuspended in binding buffer (200 μl), and sonicated on ice until the cells had clarified. Triton X-100 (1% [v/v]) was added to dissolve cell membranes, the samples were incubated on ice for 10 min, and any cell debris was removed by centrifugation (15,000*g*, 2 min, 4 °C). The cleared lysate was added to anti-FLAG agarose (25 μl) pre-equilibrated in binding buffer and incubated for 1 h at 4 °C with gentle agitation. Following incubation, the anti-FLAG agarose was washed four times with 1 ml binding buffer containing 0.1% Triton X-100 by centrifugation (1000*g*, 1 min, 4 °C). The protein was subsequently eluted with SDS-PAGE loading dye (50 μl) with heating at 80 °C. Samples were analyzed by SDS-PAGE, and Western blotting on PVDF membrane blocked with 5% nonfat skim milk. FLAG-tagged proteins were initially probed with mouse-derived anti-FLAG-HRP antibodies (1:5000 dilution) and detected by chemiluminescence. Subsequently, the HRP was inactivated with 10% H_2_O_2_ for 15 min at 37 °C. Complete inactivation was verified by reimaging the membrane. The blot was subsequently reprobed with streptavidin–HRP (1:5000 dilution) in Tris-buffered saline with 0.1% Tween-20 containing 3% bovine serum albumin and imaged by chemiluminescence.

AMA titrations were performed as aforementioned, except AMA concentrations were varied at 32, 16, 8, 4, and 2 μg⋅ml^−1^ with the appropriate MBL. In these experiments, an aliquot was taken from probed lysates before incubation with anti-FLAG beads (input) and probed separately with mouse-derived anti-FLAG-HRP and reprobed with mouse-derived anti-RNAP primary antibodies as a loading control as described previously. Semiquantitative analysis of MBL labeling by B_7_–Mal was determined using ImageLab software. The extent of labeling was normalized to the total signal from the FLAG antibody from the same respective band. Also, residual β-lactamase activity within the input samples (10 μl) was determined with nitrocefin as described in the general assay methods, and each rate was normalized to the sample with the highest activity. At least two biological and technical replicates were performed.

### Intact protein LC ESI–MS

NDM-1 (1 μM) was treated with B_7_–Mal (0.5 mM) for 5 min following its incubation (1 h) in buffer (25 mM Hepes–NaOH, 100 mM NaCl, 10 μM ZnSO_4_, and pH 7.5) either lacking or containing AMA (500 μM). The reaction mixture was incubated at 37 °C for 30 min and quenched with 1 mM DTT before LC ESI–MS analysis.

Intact protein LC ESI–MS was performed on an Agilent 6546 LC/Q-TOF in positive ion mode, with a ZORBAX StableBond 300 C3 column (Agilent; 3.0 × 150 mm, 3.5 μm) using the following method. Gas temperature of 200 °C, gas flow of 14 l/min, fragmentor: 380, buffer A: water + 0.1% formic acid, buffer B: acetonitrile + 0.1% formic acid, flow rate: 0.4 ml/min, gradient: 0 to 1 min 95% B, 1 to 4 min 5 to 38% B, 4 to 20 min 38 to 55% B, 20 to 24 min 55 to 95% B, 24 to 27 min 95% B, and 27 to 28 min 95 to 5% B. Spectra were deconvoluted, and figures were generated using UniDec software (McMarty Lab) (35).

### Peptide mapping of NDM-1

Identification of the site of biotinylation was achieved through analysis of a sequential proteolytic digest of AMA-treated NDM-1. AMA (100 μM) was added to 20 μl of NDM-1 (1 mg⋅ml^−1^) in reaction buffer. After incubation for 60 min at 37 °C, the reaction was supplemented with B7–Mal (0.5 mM) and incubated for an additional 15 min at 37 °C. A control without B7–Mal was prepared in parallel. Residual B7–Mal was eliminated with 1 mM DTT, and trypsin (2 μg) was added and incubated overnight at 37 °C. Trypsin was heat inactivated (80 °C, 20 min), and Asp-N endoproteinase (2 μg) was added and incubated for an additional 16 h at 37 °C. The digest was subsequently analyzed by LC–MS/MS using the MS system described previously. Peptides were separated on a C8 column (Agilent Eclipse XDB-C8; 100 by 2.1 mm; 3.5-μm pore size) equilibrated with 2% acetonitrile in 0.1% formic acid. Following sample injection, a linear gradient to 45% acetonitrile in 0.1% formic acid was applied over 40 min at a rate of 0.2 ml/min, followed by an increase to 55% acetonitrile over 10 min; the first 2 min of eluant was sent to waste and not the spectrometer. The mass spectrometer electrospray capillary voltage and nebulizer pressure were maintained as described previously. The fragmentor was set to 150, and nitrogen was used as both the nebulizing and drying gas and the collision-induced gas. The ions corresponding to the target peptide DNITVGIDGTDIAFGGCLIK in its modified and unmodified forms (*m*/*z* = 1011.51 [M + 2H]^2+^ and 1237.11 [M + 2H]^2+^, respectively) were identified manually and were within a mass tolerance of 5 ppm. Targeted MS/MS was performed on the modified peptide (1237.11 [M + 2H]^2+^) using a collision-induced dissociation energy of 20 with scans taken across an *m*/*z* range of 50 to 3000 in positive ion mode. Fragment ions were searched using Mmass, version 5.5.0 (www.mmass.org) with a mass tolerance of 10 ppm resulting in the identification of 25% of the total number of peaks. As we had confirmed the presence of the modification on purified recombinant protein through intact protein ESI–MS, no replicate analyses of peptide mapping was performed.

### ITC

The metal-binding titrations were performed on a MicroCal Auto-ITC200 calorimeter (Malvern). AMA, EDTA, and the metal chloride salts were dissolved in 20 mM Tris–HCl (pH 7.0) and degassed for 10 min by sonication before the experiments. The solutions of the divalent cations were titrated into AMA/EDTA solutions over 26 aliquots of 1.5 μl except for the first aliquot being 0.5 μl. The reference power was set at 2.0 μcal⋅s^−1^, and the experiment temperature was 25 °C. Blank titrations included the metal solutions titrated into the assay buffer or assay buffer titrated into AMA/EDTA solution with assay conditions identical to those described previously (all of which showed negligible signals). The experiments were performed in triplicate, and the thermograms analyzed using the manufacturer's Origin 7.0 software (OrginLab).

### ESI–MS of Zn^2+^–AMA complexes

AMA (100 μM) was incubated with an equimolar concentration of ZnSO_4_ for 10 min at room temperature. The mixture was analyzed with ESI–MS by direct infusion using a Thermo Scientific LTQ OrbiTrap XL ETD Hybrid Ion Trap-Orbitrap Mass Spectrometer (Thermo Fisher Scientific) at a flow rate of 5 μl⋅min^−1^ with a spray voltage of 4.5 kV and capillary temperature of 350 °C. The instrument was operated in Fourier transform and positive-ion modes with MS scans between 100 and 2000 *m/z*. Mass spectra were analyzed using Mmass—open source MS tool.

### Determination of Zn^2+^ affinity

The Zn^2+^ affinity of AMA was determined through Zn^2+^ competition with a colorimetric PAR-based assay. When in excess, PAR forms a 2:1 complex with Zn^2+^ ions and absorbs light at 492 nm. Dissociation of Zn^2+^ from PAR because of competition results in a decrease in absorbance at this wavelength. AMA was titrated into Chelex-100–treated buffer (20 mM Hepes, 100 mM NaCl, and pH 7.5) containing excess PAR (100 μM) and ZnSO_4_ (10 μM). Assays were incubated in clear flat-bottom 96-well plates for 3 h at 25 °C with a final assay volume of 200 μl and measured with a BioTek Synergy H1 microplate reader. For comparison, titration with EDTA was performed in parallel. As buffer conditions are known to affect both the molar extinction coefficient and stability constant of PAR, the competition assay was calibrated using EGTA (*K*_*d*_ = 623 ± 30 PM). The dissociation constant for AMA was estimated using the known stability constant of PAR at pH 7.4 using the method described by Kocyla *et al.* (23).

Determination of the Zn^2+^ affinity of NDM-1 and NDM-6 was achieved by competing for the fluorescent Zn^2+^ probes FluoZin-3 (*K*_*d*Zn_ = 9.1 nM) (36) and/or Fluo-5N (3.15 μM). The *K*_*d*Zn_ of Fluo-5N was determined by titration of ZnSO_4_ into Fluo-5N (1 μM), and the fluorescence was measured (λ_ex_/λ_em_: 494/516 nm) with a BioTek Synergy H1 microplate reader. Competition assays were performed using apoenzymes that were produced using a previously published method (13). All assays were conducted in black 96-well plates in Chelex-100–treated buffer (20 mM Hepes, 100 mM NaCl, and pH 7.5) at 25 °C in duplicate.

ZnSO_4_ was titrated into a mixture of apo-NDM-1 (2.4 μM) and FluoZin-3 (2 μM), and the fluorescence (λ_ex_/λ_em_: 494/516 nm) was measured using a BioTek Synergy H1 plate reader. The data were fit to a model describing 2:1 Zn^2+^:NDM-1 and 1:1 Zn^2+^:FluoZin-3 binding stoichiometries using DynaFit (BioKin, Ltd) (37). While competition with FluoZin-3 allowed for the accurate determination of Zn^2+^ affinity of the high binding site (Zn_1_), the affinity of the Zn_2_ site was too low to be accurately determined with this probe.

To determine the Zn^2+^ affinity of the Zn_2_ site of NDM-1, ZnSO_4_ was titrated into a mixture of apo-NDM-1 (5.5 μM) and Fluo-5N (2 μM), and the fluorescence (λ_ex_/λ_em_: 494/516 nm) was measured using a BioTek Synergy H1 plate reader. The data were fit to a model describing 2:1 Zn^2+^:NDM-1 and 1:1 Zn^2+^:Fluo-5N stoichiometry using DynaFit (Zn_1_ constant was fixed at 2.5 nM).

ZnSO_4_ was titrated into a mixture of apo-NDM-6 (4.6 μM) and FluoZin-3 (2 μM), and the fluorescence (λ_ex_/λ_em_: 494/516 nm) was measured using a BioTek Synergy H1 plate reader. The data were fit to a model describing 2:1 Zn^2+^:NDM-6 and 1:1 Zn^2+^:FluoZin-3 binding stoichiometries using DynaFit (37).

## Data availability

The authors declare that the data supporting the findings of this study are available within the article and its supporting information files. The raw MS data for the peptide analysis of biotinylated NDM-1 are available through Figshare (https://doi.org/10.6084/m9.figshare.14632722).

## Supporting information

This article contains supporting information.

## Conflict of interest

The authors declare that they have no conflicts of interest with the contents of this article.
