# Supplementary material for: Aspergillomarasmine A inhibits metallo-β-lactamases by selectively sequestering Zn2+
Source: J Biol Chem. 2021 Jun 25;297(2):100918. doi: 10.1016/j.jbc.2021.100918 (PMC8319579; doi:10.1016/j.jbc.2021.100918)
Supplement: Supplemental Figures S1–S8 and Tables S1 [file mmc1.pdf]

Supplementary Figures

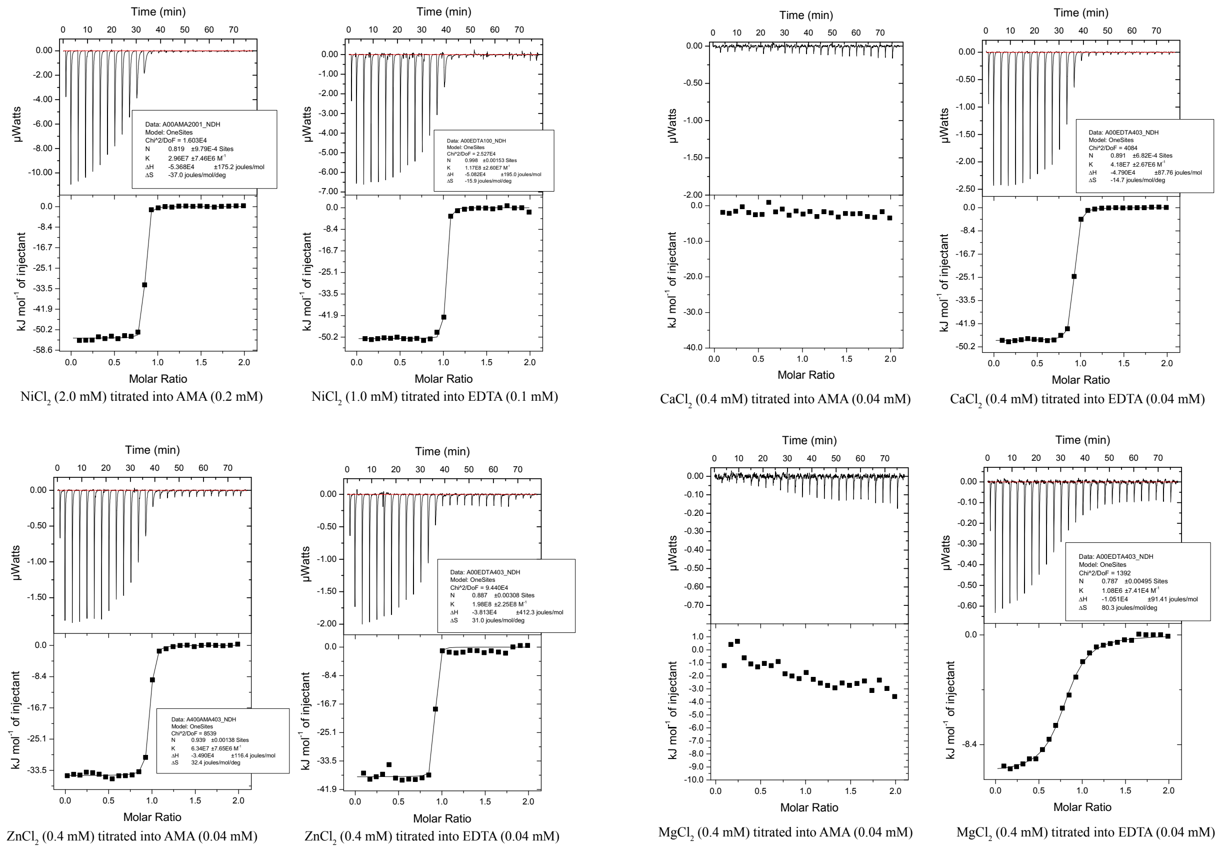

Figure S1. **ITC analysis of AMA and EDTA binding to Zn^{2+},Ni^{2+}, Mg^{2+}, and Ca^{2+}.**

Metal-binding titrations were performed as described in the Methods section. In the cases of $NiCl_2$ and $ZnCl_2$ titrated into AMA/EDTA, as well as $CaCl_2$ titrated into EDTA, the metal binding affinity was too high (K_d values estimated in low nM range) to allow an accurate determination of the binding constant. N: binding stoichiometry, K: binding constant, ΔH: enthalpy, ΔS: entropy.

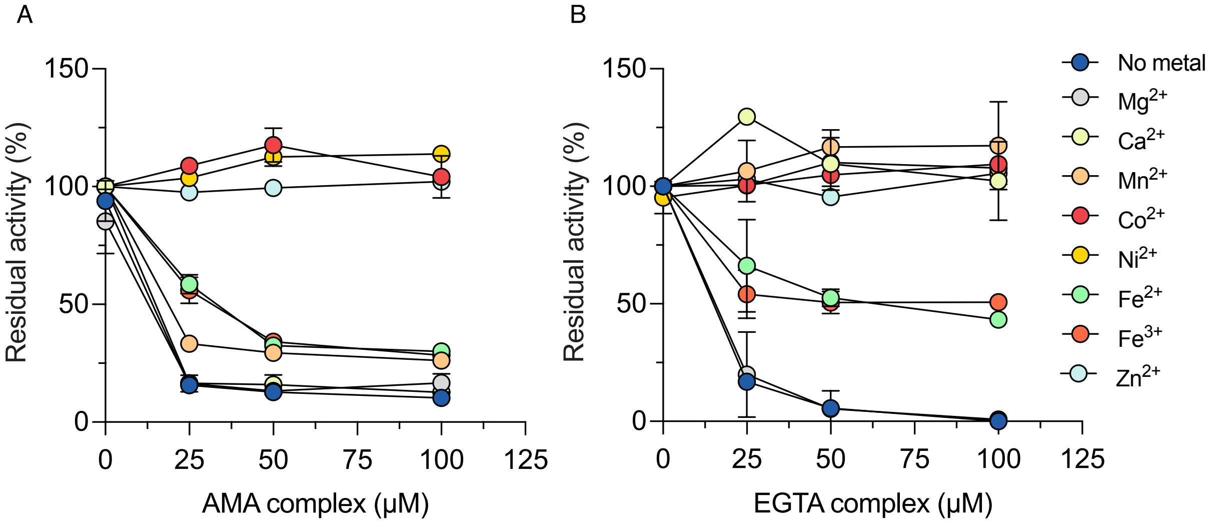

Figure S2. In vitro inhibition of recombinant NDM-1 by AMA and EGTA, and their complexes with Mg^{2+}, Ca^{2+}, Mn^{2+}, Zn^{2+}, Co^{2+}, Fe^{2+}, Fe^{3+}, and Ni^{2+}. *A*, NDM-1 (5 nM) was incubated with various concentrations of AMA mixed 1:1 with different metals (100, 50, 25, and 0 μM) in assay buffer (25 mM HEPES:NaOH, 10 μM $ZnSO_4$, 1% (*v/v*) PEG 4000, pH 7.5) at 37 ºC for 10 min. Nitrocefin (30 μM) was added to initiate the reaction and the steady state rates were measured. Residual enzyme activity was normalized to a control reaction lacking AMA. *B*, Same conditions as panel ***A***, except that EGTA was used. All data represent mean values of duplicate data points and error bars represent s.d.

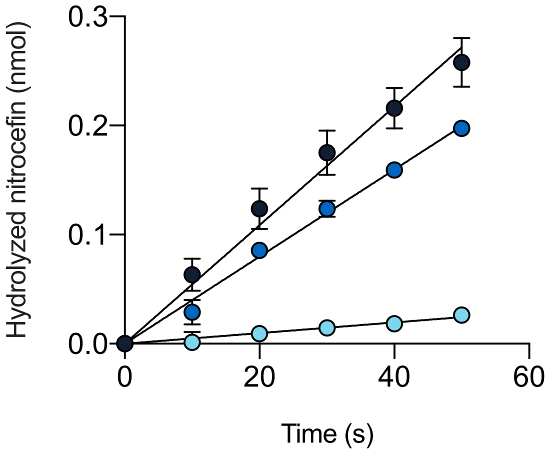

Figure S3. Reactivation of NDM-1 following spontaneous dissociation of Zn^{2+} in Chelex-100. Representative progress curves of enzyme catalyzed reactions containing NDM-1 (2 nM) in assay buffer (Chelex-100-treated 25 mM HEPES:NaOH, 1% (*v/v*) PEG 4000, 30 µM nitrocefin, pH 7.5). Control NDM-1 reaction (black) with 10 µM $ZnSO_4$, inactive NDM-1 from Chelex-treated buffer (cyan), NDM-1 reactivated with 10 µM $ZnSO_4$ (blue). Data represent mean values of duplicate data points and error bars represent s.d.

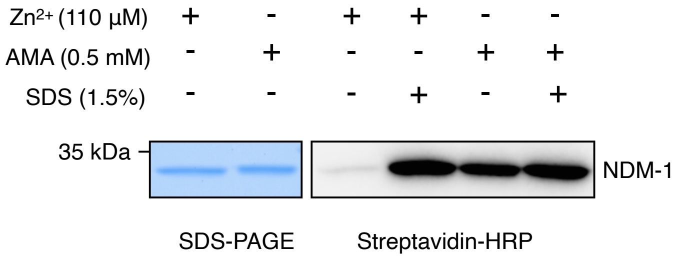

Figure S4. *In vitro* biotin-maleimide labeling of NDM-1. *In vitro* biotinylation of NDM-1 (1 µM) in assay buffer (25 mM HEPES:NaOH, 1% (*v/v*) PEG 4000, 10 µM $ZnSO_4$, pH 7.5) with (+) or without (-) AMA (0.5 mM), $ZnSO_4$ (0.11 mM), or SDS (2% *w/v*) after 30 min of incubation at 37 ºC. Left, SDS-PAGE analysis stained with Coomassie Brilliant Blue. Right, electroblot analysis with streptavidin-HRP detection shows enchanced labeling of NDM-1 (1 µM) in the presence of B_7Mal (0.5 mM) and AMA (0.5 mM). NDM-1 is not labelled in the presence of $ZnSO_4$ and entirely labelled when denatured with SDS. The data are representative of two independent experiments.

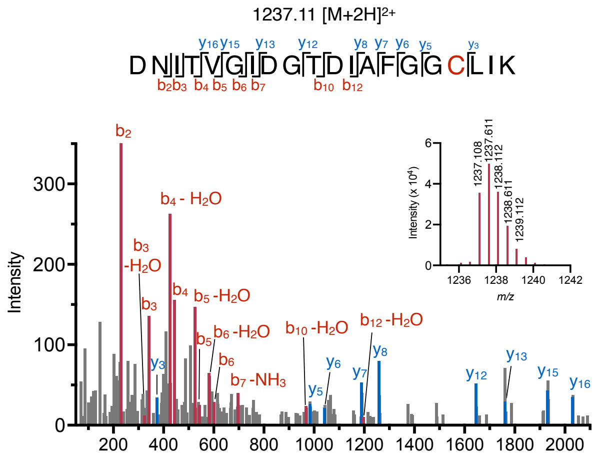

Meas. m/z	Calc. m/z	δ (ppm)	z	Annotation
230.08	230.08	0	1	b2 [1-2]
325.15	325.15	1.9	1	b3 -H2O [1-3]
343.16	343.16	-3	1	b3 [1-3]
373.28	373.28	-2.9	1	y3 [18-20]
426.2	426.2	-0.2	1	b4 -H2O [1-4]
444.21	444.21	-6.3	1	b4 [1-4]
525.26	525.27	-5	1	b5 -H2O [1-5]
543.28	543.28	-1.3	1	b5 [1-5]
582.29	582.29	9.8	1	b6 -H2O [1-6]
583.27	583.27	-6.5	1	b6 -NH3 [1-6]
600.29	600.3	-9.1	1	b6 [1-6]
696.36	696.36	-1.3	1	b7 -NH3 [1-7]
968.47	968.47	4.6	1	b10 -H2O [1-10]
984.51	984.5	9.4	1	y5 [16-20] [1xBiotin-Mal]
1041.53	1041.52	3.5	1	y6 [15-20] [1xBiotin-Mal]
1188.58	1188.59	-8.5	1	y7 [14-20] [1xBiotin-Mal]
1196.58	1196.58	2.4	1	b12 -H2O [1-12]
1259.62	1259.63	-2.7	1	y8 [13-20] [1xBiotin-Mal]
1645.8	1645.81	-1.8	1	y12 [9-20] [1xBiotin-Mal]
1760.84	1760.83	0.9	1	y13 [8-20] [1xBiotin-Mal]
1930.94	1930.94	-1.2	1	y15 [6-20] [1xBiotin-Mal]
2030.02	2030.01	3.7	1	y16 [5-20] [1xBiotin-Mal]

Figure S5. Confirmation of Cys_{208} biotinylation in NDM-1 with LC-MS/MS peptide mapping. NDM-1 (20 µg) was incubated with AMA (0.5 mM) for 1 hour followed by incubation with B_7Mal (0.5 mM) for 15 min. Unreacted B_7Mal was eliminated with 1 mM DTT and the protein was digested sequential in-solution digestion with trypsin (2 µg) and Asp-N endoproteinase (2 µg). The resultant peptides were analyzed by LC-MS/MS. Left, MS/MS spectrum of the parent ion m/z = 1237.11 $[M+2H]^{2+}$ corresponding to a biotinylated peptide. A neutral mass adduct of 451.19 Da (corresponding to biotin-maleimide modification) was identified on Cys_{208}. Right, table of corresponding m/z values and proposed sequence for each fragment ion identified.

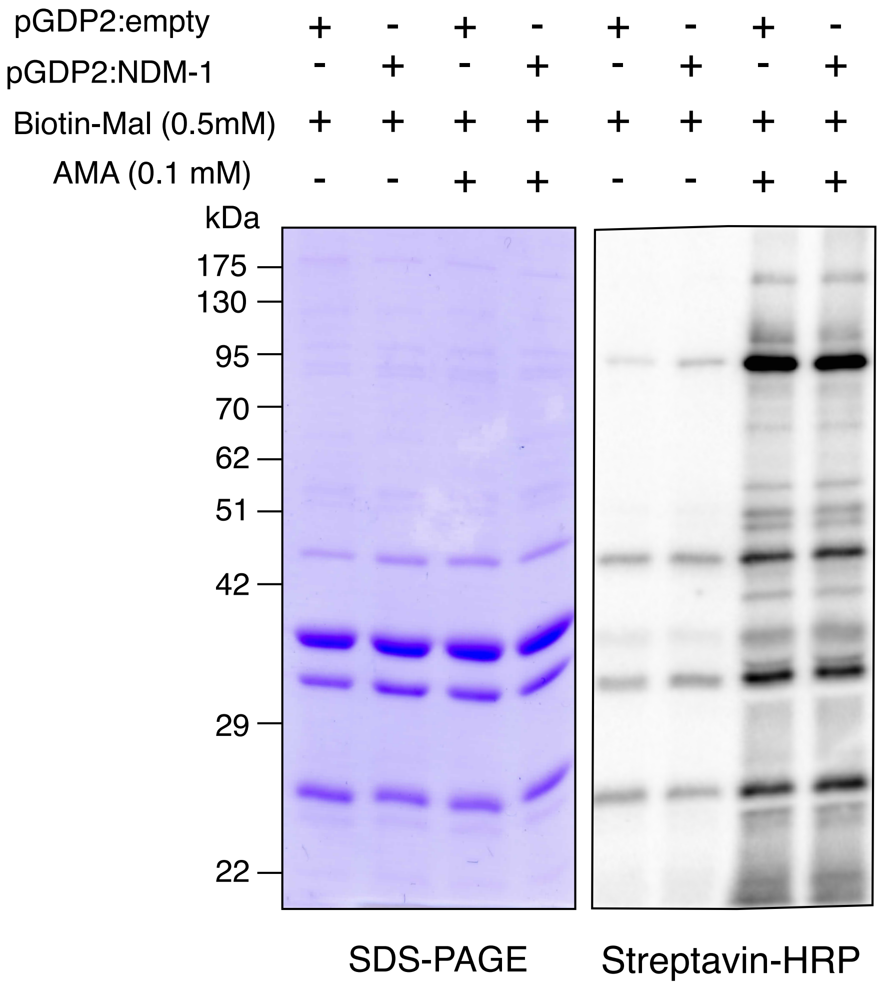

Figure S6. Streptavidin-HRP analysis of outer membrane extracts. *E. coli* with (+) or without (-) empty pGDP2 or pGDP2:NDM-1 were incubated with (+) or without (-) either AMA (0.1 mM), or B_7Mal (0.5 mM). Outer membranes were subsequently extracted and analyzed. Left, SDS-PAGE analysis of *E. coli* outer membranes. Right, electroblot analysis of the panel on the left probed with streptavidin-HRP.

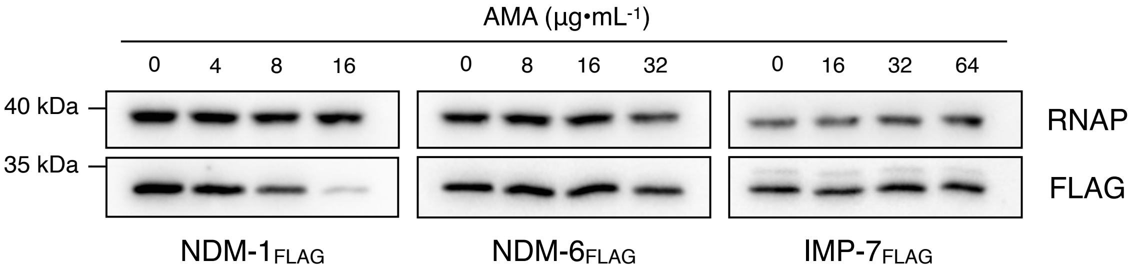

Figure S7. **Immunoblot analysis of NDM-1$_{FLAG}$, NDM-6$_{FLAG}$, and IMP-7$_{FLAG}$ in whole cells.** *E. coli* producing each respective MBL were treated with various concentrations of AMA for 30 min at 37 ºC in CAMHB. Cells were lysed in SDS-PAGE loading dye and analyzed by immunoblotting with anti-FLAG antibodies. Anti-RNAP antibodies were used as a loading control.

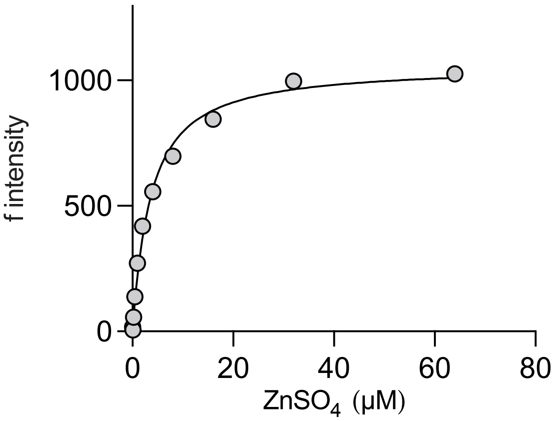

Figure S8. Zn^{2+} affinity of Fluo-5N. Fluorescence response of Fluo-5N (1 μM) to direct titration of $ZnSO_4$ in 20 mM HEPES:NaOH, 100 mM NaCl, pH 7.5. The solid black line represents the fit to a 1:1 Zn^{2+}:Fluo-5N model.

Supplementary Table 1. **MIC values for meropenem against *E. coli* BW25113 harbouring different MBLs. MBL genes were expressed from the pGDP2 plasmid[a]**

Strain	Plasmid	Meropenem (μg·mL^{-1})
***E. coli* BW25113**	NDM-1	32
	NDM-6	64
	IMP-7	16
	NDM-1$_{FLAG}$	32
	NDM-6$_{FLAG}$	64
	IMP-7$_{FLAG}$	16

[a]Determined in CAMHB in duplicate
